# Effects of different Lys/Met ratios on the antioxidant capacity, tissue morphology, and fatty acid composition of subcutaneous fat in Tibetan sheep on low-protein diets: a lipidomic analysis

**DOI:** 10.3389/fvets.2024.1528331

**Published:** 2025-01-30

**Authors:** Rengeerli Sa, Fengshuo Zhang, Xianhua Zhang, Wei Gao, Yu Zhang, Jiacheng Gan, Shengzhen Hou, Linsheng Gui

**Affiliations:** College of Agriculture and Animal Husbandry, Qinghai University, Xining, China

**Keywords:** lipidomics, subcutaneous fat, low protein, amino acids, nutritional regulation

## Abstract

**Introduction:**

This study employed lipidomics to investigate the effects of varying lysine (Lys)- to-methionine (Met) ratios on the antioxidant capacity, tissue morphology, and fatty acid composition of subcutaneous fat in Tibetan sheep fed a low-protein diet.

**Methods:**

Ninety healthy male Tibetan sheep of similar body weight were randomly allocated into three groups. These sheep were fed a low-protein diet containing Lys/Met ratios of 1:1, 2:1, and 3:1. Ultra-High Performance Liquid Chromatography–tandem Mass Spectrometry (UHPLC–MS/MS) was employed to explore the changes in various lipid subclasses in subcutaneous adipose tissue. The expression of genes associated with adipogenesis, antioxidant capacity, and fatty acid metabolism was also examined.

**Results:**

The results indicated that the 1:1 Lys/Met group exhibited significantly higher antioxidant capacity (glutathione peroxidase, GSH-Px), with more orderly adipocyte arrangement, uniform cell size, and a general increase in unsaturated fatty acid levels. Additionally, several lipid molecules associated with the phenotype (Antioxidant index and fatty acid content) were identified, namely, DG(38:3e) + Na, PE(17:1_22:2)-H, PI(17:0_20:3)-H, TG(33:0e) + NH4, Cer(d14:0_17:1) + H, and CL(81:13)-2H. Furthermore, the findings showed that the upregulation of *PPARγ, FASN, FAD4, CPT1A*, and *GPX4* can enhance adipocyte differentiation and lipid accumulation, thereby improving metabolic function in subcutaneous adipose tissue via the regulation of lipid metabolism and oxidative defense mechanisms.

**Discussion:**

In summary, this study provides a theoretical foundation for optimizing precision feeding strategies for Tibetan sheep, offering crucial data to support enhancements in production efficiency and meat quality.

## 1 Introduction

Protein feed plays an essential role in animal breeding, supporting the healthy growth of animals, increasing breeding and production efficiency, and enabling environmental protection (1). However, when animals are fed diets high in protein, the protein content often exceeds their physiological needs, leading to resource wastage, increased production costs, and reduced economic efficiency (2). To address these issues, low-protein diets supplemented with exogenous amino acids have emerged as a key component of precision feeding and are gaining acceptance among farmers and breeders (3). Studies have shown that moderately lowering the protein content of the diet while exogenously providing amino acids can provide significant benefits and improve host immunity, gut microbiota, intestinal health, and nitrogen utilization efficiency (4). Among the various essential amino acids (EAAs), lysine (Lys) and methionine (Met) which are important components of proteins are considered the most crucial limiting amino acids in ruminants (5). In fact, studies have demonstrated that dietary Met and Lys are the most important EAAs for growing lambs owing to their roles in promoting protein synthesis and regulating lipid metabolism (6). However, the optimal Lys/Met ratio necessary to achieve an amino acid balance in low-protein diets is yet to be determined.

In addition to serving as a primary reservoir for energy, mammalian adipose tissue is also a metabolically active organ that is closely involved in overall metabolic regulation (7). In livestock living on the Tibetan Plateau, subcutaneous fat is a type of adipose tissue, acts as a key fat reservoir and contributes to immune defense and mechanical protection (8). The composition and distribution of subcutaneous fat also affects meat quality in lambs, since the right amount of subcutaneous fat increases the tenderness, juiciness, and flavor of the meat (9). During prolonged periods of starvation or illness, subcutaneous fat is broken down into fatty acids (FAs) and glycerol to provide the body with essential nutrients (10). Therefore, studying the content and composition of subcutaneous fat is essential for developing feeding strategies aimed at improving economic efficiency in animal breeding.

The composition of adipose tissue can be analyzed using various methods. For instance, an enzyme-linked immunosorbent assay (ELISA) can be used to measure the concentration of malondialdehyde (MDA) and examine the levels of glutathione peroxidase (GSH-Px), catalase (CAT), and superoxide dismutase (SOD) in adipose tissue. Meanwhile, FA components in adipose tissue can be revealed using ultra performance liquid chromatography-mass spectrometry/mass spectrometry (GC–MS). Lipidomics involves a systematic evaluation of the types of lipid molecules present in a particular sample, their structures, their functions, and their interactions. This method can elucidate the diversity of lipid molecules and accurately reveal their chemical structures through the application of high-resolution MS and nuclear magnetic resonance (NMR) technologies, delineating the distribution and proportion of different types of lipids (11).

Tibetan sheep, a primitive breed native to the Tibetan Plateau, are notable for their resistance to cold environments and adaptability to rough grazing conditions and are thus a vital part of the alpine livestock industry (12). In this study, we investigated the effects of different Lys/Met ratios on subcutaneous fat tissue in Tibetan sheep reared on low-protein diets by analyzing the antioxidant capacity, tissue morphology, and fatty acid composition. This study sought to provide data support for optimizing dietary allocations and enhancing economic efficiency during the breeding of Tibetan sheep while also improving meat quality.

## 2 Materials and methods

### 2.1 Experimental conditions and diet

The trial was conducted from April to July 2022 at Jinzang Ranch, Haiyan County, Haibei Tibetan Autonomous Prefecture, Qinghai Province, China. The total duration of the trial was 100 days, which included a 10-day pre-feeding period and a 90-day main trial phase. Ninety healthy, disease-free male lambs (plateau-type Tibetan sheep) with similar body weights (15.73 ± 0.90 kg) were selected for the experiment and randomly divided into three groups of 30 lambs: LP-L (Lys/Met 1:1), LP-M (Lys/Met 2:1), and LP-H (Lys/Met 3:1). Each group consisted of five replicates, with six lambs per replicate. At the end of the experiment, three lambs were randomly selected from one replicate of each group, making a total of nine lambs slaughtered across the three groups. The experimental diet comprised 70% concentrate and 30% roughage, with a 1:1 dry matter ratio of oat silage to oat hay. The protein content of the concentrate diet was 10%, with the Lys/Met ratio varying among the groups. The feed formulation is shown in Table 1.

**Table 1:** Dietary concentrate composition and nutrient levels (dry matter basis).

Items	LP-L	LP-M	LP-H
Ingredient (%)			
Oat hay	15.00	15.00	15.00
Oat silage	15.00	15.00	15.00
Corn	36.53	37.10	37.10
Wheat	7.70	7.70	7.70
Soybean meal	0.70	0.70	0.70
Rapeseed meal	7.00	7.00	7.00
Cottonseed meal	0.70	0.70	0.70
Maize germ meal	0.70	0.70	0.70
Palm meal	11.20	11.20	11.20
NaCl	0.35	0.35	0.60
Limestone	0.35	0.44	0.70
Baking soda	0.07	0.00	0.07
Premix^a^	2.94	2.94	2.94
Lys	1.39	0.93	0.48
Met	0.37	0.24	0.11
Total	100.00	100.00	100.00
Nutrient levels			
DE (MJ/kg)^b^	10.76	10.84	10.84
Crude protein	9.94	9.98	9.98
Ether extract	2.85	2.87	2.87
Crude fiber	22.47	22.61	22.61
Neutral detergent fiber	33.72	33.77	33.77
Acid detergent fiber	23.37	23.39	23.39
Ca	0.42	0.42	0.42
P	0.17	0.17	0.17

a Provided per kilogram of diets: Cu 15 mg, Fe 55 mg, Zn 25 mg, Mn40 mg, Se 0.30 mg, I 0.5 mg, Co 0.20 mg, VA 20000 IU, VD 4000 IU, VE 40 IU.

b Digestive energy is the calculated value, while the rest are measured values.

At the end of the trial, the test sheep were euthanized according to the ARRIVE guidelines (American Veterinary Medical Association Guidelines for Animal Euthanasia: 2020 Edition). Pentobarbital Sodium was injected through the jugular vein at 80–100 mg/kg to ensure smooth entry of the drug into the sheep’s body. After injection, the sheep were usually unconscious within 30 s to 1 min, died within 3–5 min, and were slaughtered after confirmation of death by the absence of corneal reflexes, confirmation of the absence of a heartbeat by a stethoscope, and fixation and dilatation of the pupils. Subcutaneous adipose tissue from the back of Tibetan sheep was collected, and part of it was fixed in paraformaldehyde solution for histomorphometric testing. One part was frozen in liquid nitrogen and stored at −80°C for subsequent lipid metabolism testing.

### 2.2 Determination of the antioxidant capacity of fat

First, 1.0 g of subcutaneous tissue was weighed and transferred to a centrifuge tube. Then, 0.9 mL of phosphate-buffered saline (PBS) was added, and the mixture was homogenized using a tissue pulverizer (Tiss-Basic48, Shanghai Jingshen, China) to obtain a turbid liquid. The homogenate was centrifuged at 3,000 × g for 20 min, and the supernatant was stored at −20°C. Antioxidant indexes, including catalase (CAT), superoxide dismutase (SOD), glutathione peroxidase (GSH-Px), and malondialdehyde (MDA) in subcutaneous fat was evaluated using a one-step sandwich enzyme-linked immunosorbent assay (ELISA).

### 2.3 Observation of adipose tissue morphology

Subcutaneous tissues were collected from Tibetan sheep, fixed in paraformaldehyde, and embedded in paraffin. Tissue sections were stained with hematoxylin and eosin (HE). The microscope used for HE staining was an orthogonal light microscope (Nikon Eclipse E100). The diameter of adipocytes was observed and calculated. At least 10 adipocytes were observed per section.

### 2.4 Fatty acid composition analysis

An 8890-7000D Gas Chromatography/Mass Selective Detector (GC/MSD) gas-mass spectrometer (Agilent Technologies, CA, United States) was used to analyze FA composition with an Agilent DB-FastFAME capillary column (20 m, 0.18 mm, 0.2 μm; Agilent J&W Scientific, Folsom, CA, United States). High-purity helium (purity not less than 99.999%) was used as the carrier gas, with a flow rate of 1.0 mL/min. The injection port temperature was 230°C, the injection volume was 1 μL, and the sample was injected through a shunt, with a ratio of 50:1 and a solvent delay of 1.0 min. The initial column temperature was 80°C, maintained for 0.5 min, then increased to 175°C at a rate of 70°C/min, and finally to 230°C at a rate of 8°C/min. The temperature was maintained at 230°C for 1 min. The column temperature was then decreased to 80°C and maintained for 2 min. The mass spectrometry conditions were as follows: electron ionization (EI), ion source temperature of 230°C, four-stage rod temperature of 150°C, transmission line temperature of 240°C, and electron energy of 70 eV. The ion scanning mode (SIM) was selected.

### 2.5 Lipidomic analysis

A suitable amount of sample was mixed with 200 μL of water and 20 μL of an internal lipid standard. The mixture was vortexed, followed by the addition of 800 μL of MTBE and 240 μL of pre-cooled methanol. The mixture was vortexed again, sonicated for 20 min in a low-temperature water bath, and left at room temperature for 30 min. Subsequently, the mixture was centrifuged at 14,000 × g and 10°C for 15 min. The upper organic phase was removed and dried under nitrogen. For mass spectrometry analysis, 200 μL of a 90% isopropanol/acetonitrile solution was added to the supernatant, and the mixture was vortexed. Then, 90 μL of the mixture was centrifuged at 14,000 × g and 10°C for 15 min, and the supernatant was used for further LC–MS/MS analysis.

Chromatographic analysis: Samples were separated using a Ultra-High Performance Liquid Chromatography (UHPLC) Nexera LC-30A system equipped with a C18 column at a flow rate of 300 μL/min. Chromatographic analysis: Samples were separated using a UHPLC Nexera LC-30A system equipped with a C18 column at a flow rate of 300 μL/min. The first proportion of the mobile phase was maintained at 30% for 0–2 min, increased linearly from 30 to 100% for 2–25 min, and then maintained at 30% for 25–35 min. Throughout the analysis, the samples were placed in an autosampler at 10°C and analyzed consecutively in a random order.

Mass spectrometry: Electrospray ionization (ESI) was performed in both positive and negative ion modes. The samples were separated by UHPLC and analyzed via MS using a Q Exactive series mass spectrometer (Thermo Scientific™). The heater temperature was 300°C, sheath gas flow rate, 45 arb, aux gas flow rate, 15 arb, sweep gas flow rate, 1 arb, spray voltage, 3.0 kV, capillary temperature, 350°C, S-Lens RF level, 50%, and MS1 scan range, 200–1800. The mass-to-charge ratios of lipid molecules and their fragments were analyzed by collecting 10 fragmentation profiles (MS2 scan, HCD) after each full scan. The MS1 and MS2 resolutions at m/z 200 were set to 70,000 and 17,500, respectively.

### 2.6 Quantitative PCR analysis of adipose tissue RNA

Total RNA was extracted from adipose tissue using the Total RNA Kit (TaKaRa, Dalian, China) and reverse transcribed using the PrimeScript™ RT Reagent Kit (TaKaRa, Dalian, China). Quantitative PCR (qPCR) was performed on the Applied Biosystems 7,500 Fast Real-Time PCR System (Applied Biosystems, United States). Relative expression levels were calculated by the 2^ΔΔCt^ method, with *GAPDH* serving as the internal reference gene. Details of the primer sequences, annealing temperatures, and PCR product lengths are given in Table 2.

**Table 2:** Primers used in qRT-PCR.

Name	Primer sequence (5′-3′)	Tm (°C)	Product length
*PPARγ*	F-GAGCCTGCGAAAGCCCTTTG	60.0	80 bp
	R-CATCTAATTCCAGTGCGTTGAACTTC		
*FOXO1*	F-CCTACGCCGACCTCATCACC	60.0	93 bp
	R-GCACGCTCTTGACCATCCAC		
*LEP*	F-GGGTCACTGGTTTGGACTTCATC	60.0	98 bp
	R-ACTGGCGAGGATCTGTTGGTAG		
*PLIN1*	F-GAGGAGACCGAGGAGGAGGAG	60.0	99 bp
	R-CGCCATGCTGCCCAGGAG		
*FASN*	F-AGATGAAGGTGGTAGAGGTGCTAG	60.0	112 bp
	R-TGGCGGTCAGTGGCTATGTAG		
*CPT1A*	F-TCACATCCAGGCGGCAAGAG	60.0	119 bp
	R-AGCAGAGCGGAATCGTAGACC		
*FAD4*	F-GCCCTACAACTACCAGCACAAATAC	60.0	113 bp
	R-CCACCCACTTCTTCCTCTTGATAAC		
*ADIPOQ*	F-ACCACTATGACGGCACCACTG	60.0	100 bp
	R-CTGACCTTCACATCCTTCAAGTAGAC		
*GPX4*	F-GACGACGCCCACCCTCTG	60.0	116 bp
	R-ACACAGCCGTTCTTATCAATCAGG		
*GAPDH*	F-ACCTGCCGCCTGGAGAAAC	60.0	104 bp
	R-TGGTCCTCAGTGTAGCCTAGAATG		

### 2.7 Statistical analysis

Data were analyzed with SPSS version 26.0. Continuous, normally distributed data were expressed as mean ± standard error of the mean (SEM). Differences among the groups were analyzed by one-way ANOVA, followed by *post hoc* analysis with Duncan’s test. Statistical significance was defined as *p* < 0.05.

## 3 Results and analysis

### 3.1 Antioxidant indicators

The concentration of GSH-Px was significantly higher in the LP-L group than in the LP-H and LP-M groups (*p* < 0.05), but there was no significant difference in the concentration of GSH-Px between the LP-H and LP-M groups (*p* > 0.05) (Table 3).

**Table 3:** Determination of antioxidant index.

Items	LP-H	LP-M	LP-L	*p*-value
MDA (nmol/ml)	3.18 ± 0.51	2.96 ± 0.14	1.36 ± 0.53	0.230
SOD (U/ml)	79.96 ± 10.11	96.47 ± 4.97	95.94 ± 11.26	0.328
GSH-Px (U/ml)	126.59 ± 4.42^b^	129.76 ± 5.99^b^	172.62 ± 9.11^a^	0.038
CAT (U/L)	6.90 ± 0.19	4.08 ± 1.32	7.66 ± 1.28	0.094

a, b Means with different superscripts in the same row are significantly different (*p* < 0.05). Data are presented as mean ± SEM. LP-H, supplemented with a low-protein diet with a Lys/Met ratio of 3:1; LP-M, supplemented with a low-protein diet with a Lys/Met ratio of 2:1; LP-L, supplemented with a low-protein diet with a Lys/Met ratio of 1:1. MDA, malondialdehyde; SOD, superoxide dismutase; GSH-Px, glutathione peroxidase; CAT, catalase; GSH-Px, glutathione peroxidase; CAT, catalase.

### 3.2 Adipose tissue morphology

**Figure 1:**
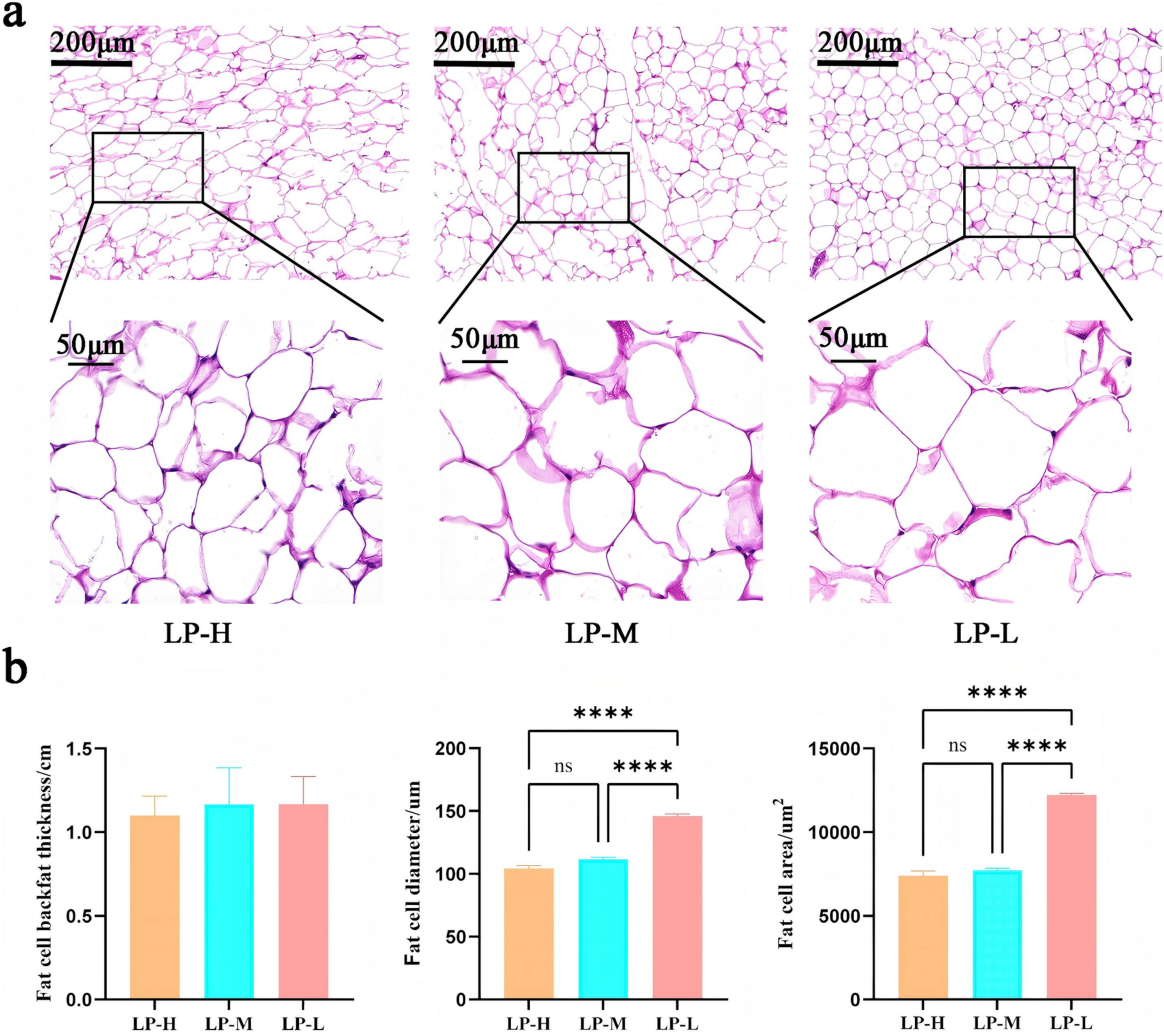
Morphologic results of adipose tissue. **(a)** Adipose backfat thickness, adipocyte diameter and adipocyte area maps of subcutaneous fat among the three groups. **(b)** HE sections of adipocytes of the three groups at 200 μm and 50 μm field of view.

Observation of HE-stained sections revealed that the adipocytes in the LP-L group were more neatly arranged than those in the LP-H and LP-M groups, with a uniform overall size and rhombic shape. In contrast, the adipocytes in the LP-H and LP-M groups were tightly arranged, and most of them were oval in shape. Additionally, some adipocytes in these groups were irregularly arranged in a scattered manner (Figure 1A). Compared with the adipocytes in the LP-L group, the adipocytes in the LP-H and LP-M groups were significantly smaller in diameter and volume (*p* < 0.05). However, there was no significant difference in the thickness of backfat among the three groups (*p* > 0.05) (Figure 1B).

### 3.3 Fatty acid composition

Among the saturated FAs, tridecanoic acid C13:0 and pentadecanoic acid C15:0 showed significantly lower levels in the LP-L group than in the LP-M and LP-H groups (*p* < 0.05). Meanwhile, the contents of undecanoic acid C11:0 and heptadecanoic acid C17:0 were significantly higher in the LP-H group than in the LP-M group (*p* < 0.05). Among the unsaturated FAs, eicosadienoic acid C20:2, eicosapentaenoic acid C20:5n3, and ginkgolic acid C17:1 exhibited significantly higher levels in the LP-L group than in the LP-H group (*p* < 0.05) (Table 4).

**Table 4:** Determination of medium and long chain fatty acid content.

Items	LP-H	LP-M	LP-L	*p*-value
SFA				
C6:0	0.02 ± 0.01	0.03 ± 0.01	0.05 ± 0.01	0.054
C10:0	9.62 ± 0.17	9.45 ± 0.74	9.49 ± 0.42	0.082
C11:0	0.22 ± 0.01^b^	0.39 ± 0.03^a^	0.03 ± 0.01^b^	0.002
C12:0	28.35 ± 0.61	24.07 ± 2.19	22.44 ± 0.94	0.061
C13:0	1.29 ± 0.04^b^	1.80 ± 0.15^a^	1.16 ± 0.04^b^	0.006
C14:0	17.21 ± 0.39	17.79 ± 1.46	11.76 ± 0.40	0.080
C15:0	32.25 ± 1.09^b^	34.02 ± 4.64^b^	40.24 ± 1.34^a^	0.029
C16:0	1951.70 ± 45.42	1992.06 ± 131.21	1886.68 ± 52.06	0.696
C17:0	87.80 ± 2.62^b^	115.16 ± 6.29^a^	103.46 ± 3.38^ab^	0.013
C18:0	1534.87 ± 49.22	1608.63 ± 113.64	1828.15 ± 51.01	0.082
C20:0	28.64 ± 1.49^b^	25.55 ± 1.82^b^	31.05 ± 1.51^a^	0.015
C21:0	1.00 ± 0.04	0.90 ± 0.09	1.12 ± 0.05	0.111
C22:0	3.70 ± 0.10	3.10 ± 0.15	3.79 ± 0.15	0.052
C23:0	1.61 ± 0.01	1.63 ± 0.03	1.69 ± 0.02	0.063
C24:0	1.60 ± 0.02	1.66 ± 0.05	1.75 ± 0.04	0.088
UFA				
C14:1	459.08 ± 10.17	444.04 ± 37.77	389.43 ± 13.05	0.093
C15:1	4.64 ± 0.18	3.94 ± 0.33	3.90 ± 0.16	0.077
C16:1	221.11 ± 4.97	226.22 ± 16.86	190.24 ± 5.83	0.062
C17:1	47.02 ± 1.26^b^	68.70 ± 5.01^a^	62.36 ± 1.46^a^	0.005
C18:1n9c	2740.28 ± 12.66	2827.03 ± 193.50	2898.96 ± 68.63	0.525
C18:2n6t	28.47 ± 0.65	31.71 ± 1.24	29.35 ± 0.64	0.062
C18:2n6C	149.18 ± 3.42	149.73 ± 12.46	125.10 ± 3.84	0.110
C18:3n6	2.24 ± 0.06	2.31 ± 0.09	2.13 ± 0.04	0.228
C18:3n3	22.06 ± 0.74	21.12 ± 1.50	18.02 ± 0.50	0.067
C20:1n9	56.54 ± 1.17	63.43 ± 3.93	57.90 ± 0.29	0.175
C20:2	4.93 ± 0.10^b^	6.69 ± 0.55^a^	6.43 ± 0.13^a^	0.019
C20:3n6	2.51 ± 0.05	2.74 ± 0.17	2.66 ± 0.06	0.388
C20:4n6	4.69 ± 0.11	6.03 ± 0.50	5.54 ± 0.11	0.051
C20:3n3	1.05 ± 0.03	1.17 ± 0.02	1.11 ± 0.03	0.051
C20:5n3	0.61 ± 0.05^b^	0.91 ± 0.09^a^	0.94 ± 0.06^a^	0.045
C22:1n9	25.36 ± 0.94	26.19 ± 1.98	22.15 ± 0.96	0.173
C22:2n6	1.42 ± 0.15	1.97 ± 0.23	1.49 ± 0.04	0.093

a, b Means with different superscripts in the same row are significantly different (*p* < 0.05). Data are presented as mean ± SEM. LP-H, supplemented with a low-protein diet with a Lys/Met ratio of 3:1; LP-M, supplemented with a low-protein diet with a Lys/Met ratio of 2:1; LP-L, supplemented with a low-protein diet with a Lys/Met ratio of 1:1. SFA, saturated fatty acids; UFA, unsaturated fatty acid.

### 3.4 Lipidomics analysis

#### 3.4.1 Model quality validation

**Figure 2:**
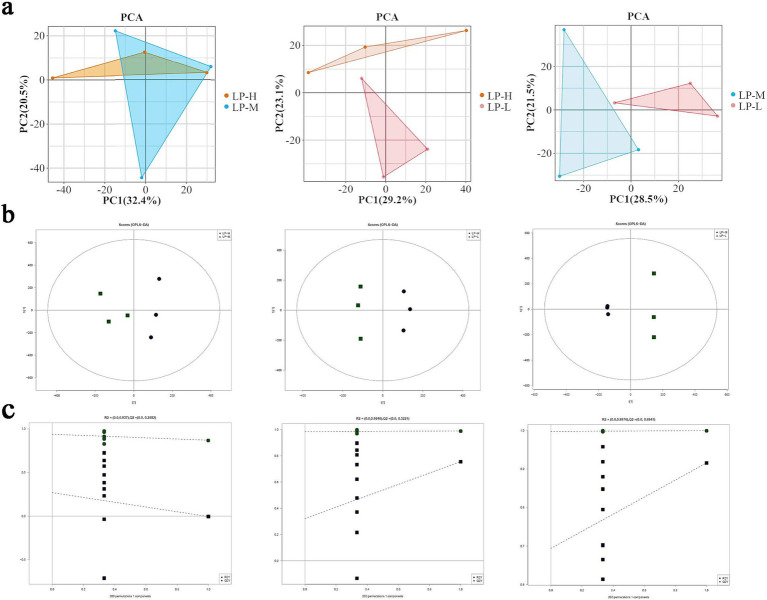
Model quality validation based on LC–MS/MS data: **(A)** PCA plots for the LP-H, LP-M, and LP-L groups; **(B)** OPLS-DA score plots for the LP-H, LP-M, and LP-L groups; **(C)** Permutation test plots for the OPLS-DA models.

In this study, we attempted to use lipidomics to investigate the changes in lipid molecules associated with subcutaneous fat in Tibetan sheep under low-protein diets containing different Lys/Met ratios. The identified lipid molecules were subjected to principal component analysis (PCA). Interestingly, the three groups showed different clustering trends on the PCA score plot, which indicated that there were significant differences in lipid composition among these groups (Figure 2A). The orthogonal partial least squares-discriminant analysis (OPLS-DA) score plot showed that when pairwise comparisons were performed among the three groups of samples, each pair of groups showed a distinct separation. This suggested that different Lys/Met radios could significantly change endogenous lipid levels in the subcutaneous fat of Tibetan sheep (Figure 2B). To avoid overfitting in the OPLS-DA model, a replacement test was performed for validation. Seven cycles of interactive validation with the replacement test showed that the OPLS-DA model was of good quality (Figure 2C).

**Figure 3:**
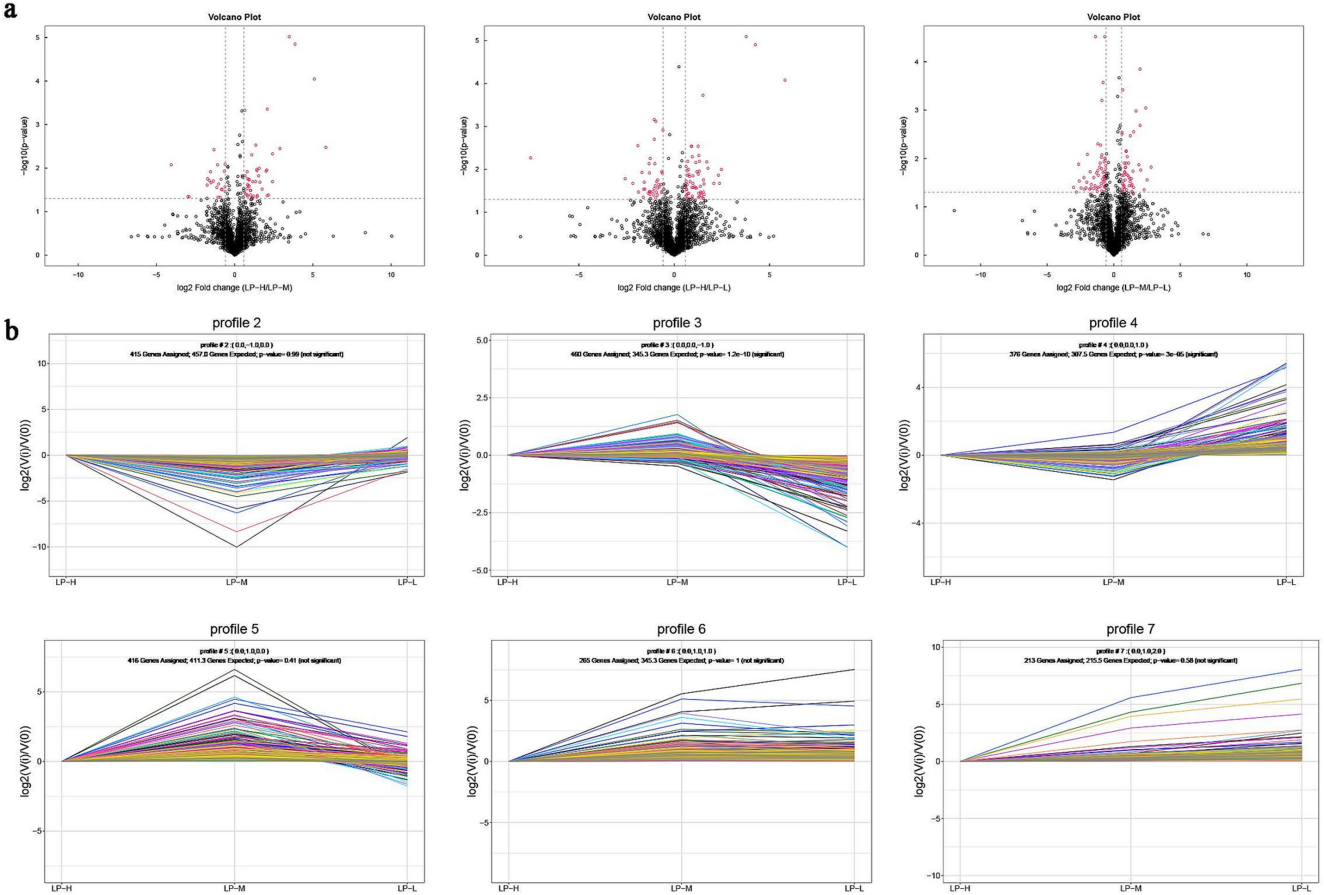
Differential lipid analysis: **(A)** Volcano plots comparing the LP-H vs. LP-M, LP-H vs. LP-L, and LP-M vs. LP-L groups (rose: significantly different lipid molecules; black: non-significantly different lipid molecules); **(B)** Trend analysis plots for lipid profiles 2–7 (each dot represents a sample, lines represent changes in lipid molecules between samples, and different colored lines represent different lipid molecules).

Volcano plots revealed that the relationship between adjusted *p*-value and subcutaneous fat lipid changes was significant (*p* < 0.05). It also revealed the presence of 376 differentially expressed lipid molecules, including 94 in the LP-H vs. LP-M pairwise comparison, 132 in the LP-H vs. LP-L pairwise comparison, and 150 in the LP-L vs. LP-M pairwise comparison. All these tests were indicative of significant differences in the lipid composition of subcutaneous fat among the three groups of samples (Figure 3A). Trend analysis plots illustrated the different abundance trends of lipid molecules under different Lys/Met feed ratios, with Profile3 and Profile4 emerging as significant (Figure 3B).

#### 3.4.2 Lipid subclass composition

**Figure 4:**
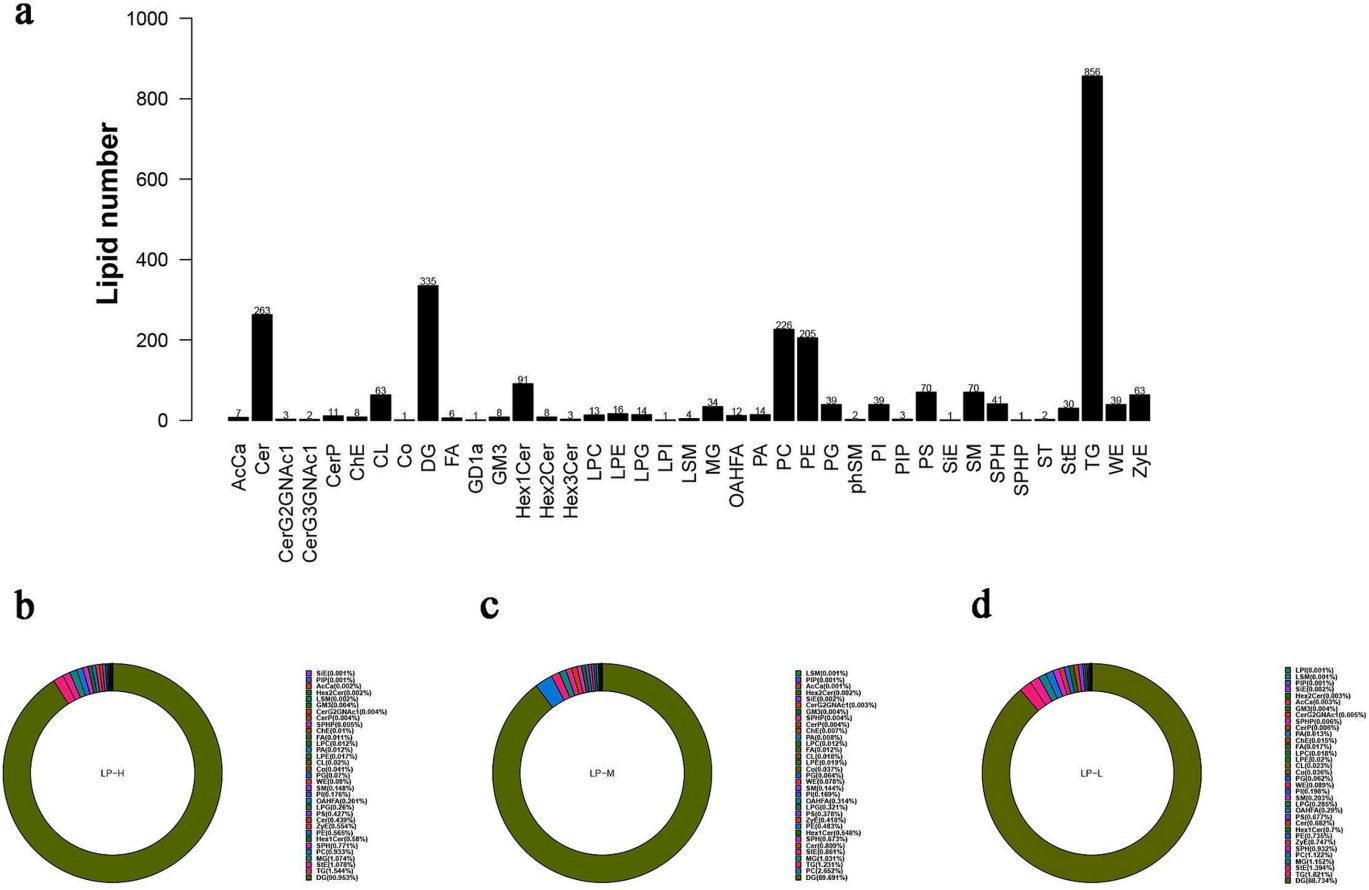
Analysis of lipidomics results. **(A)** The number of lipid subclasses and lipid molecules identified through positive and negative ion modes. **(B)** Composition distribution map of LP-H lipid subclasses **(C)** Composition distribution diagram of LP-M lipid subclasses **(D)** Composition distribution map of LP-L lipid subclasses.

The differential metabolites in the subcutaneous fat of Tibetan sheep were screened based on the OPLS-DA model. A total of 39 lipid subclasses were identified in the samples under the positive and negative ion models, with a total of 2,605 lipid molecules identified in each category (Figure 4A). Lipid composition analysis revealed that the top 10 lipid subclasses in the LP-L group were DG (88.734%), TG (1.821%), StE (1.394%), MG (1.152%), PC (1.122%), SPH (0.932%), ZyE (0.747%), PE (0.735%), Hex1 Cer (0.7%), and Cer (0.682%). Meanwhile, the top 10 lipid subclasses in the LP-H group were DG (90.953%), TG (1.544%), StE (1.078%), MG (1.074%), PC (0.933%), SPH (0.771%), Hex1 Cer (0.58%), PE (0.565%), ZyE (0.554%), and Cer (0.439%). Finally, the top 10 lipid subclasses in the LP-M group were DG (89.691%), PC (2.652%), TG (1.231%), MG (1.031%), stE (0.861%), Cer (0.809%), SPH (0.673%), HeX 1Cer (0.548%), PE (0.483%), and zyE (0.418%) (Figure 4B). Notably, the proportion of TG, StE, MG, SPH, ZyE, PE, and Hex1 Cer was higher in the LP-L group than in the LP-H and LP-M groups, while that of DG was lower. This suggested that the percentage of subcutaneous fat containing glycerol decreased when the ratio of amino acids was altered.

#### 3.4.3 Identification of significantly different lipid molecules

**Figure 5:**
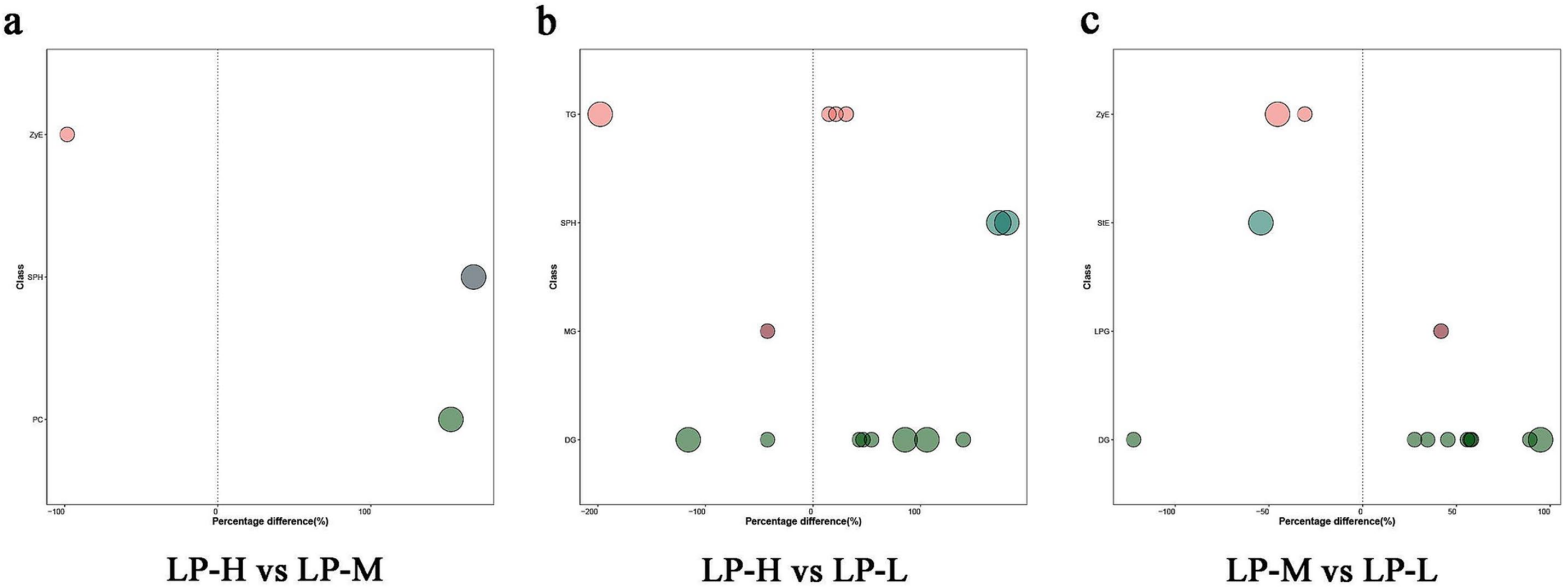
Significantly different lipid molecules between treatment groups. Bubbles represent lipid molecules with significant differences between **(A)** LP-H and LP-M, **(B)** LP-H and LP-L, and **(C)** LP-M and LP-L. The vertical axis indicates lipid subclasses, distinguished by colors. Bubble size represents the significance of the difference, with smaller bubbles indicating significant differences (0.01 < *p* < 0.05) and larger bubbles indicating highly significant differences (*p* < 0.01). The horizontal axis shows the percentage difference in lipid molecule levels between the compared groups.

Based on the relevant screening criteria of projected importance of variables values (VIP, VIP > 1) and *p*-values (*p* < 0.05), 11 lipid subclasses with significant differences were screened. Among them, three (ZyE, SPH, and PC) lipid subclasses showed significant differences between the LP-H and LP-M groups, four (DG, MG, SPH, and TG) showed significant differences between the LP-H and LP-L groups, and four (DG, LPG, StE, and ZyE) showed significant differences between the LP-M and LP-L groups. Among them, the lipid molecules screened for phenotypic correlation were DG(38:3e) + Na, PE(17:1_22:2)-H, PI(17:0_20:3)-H, TG(33:0e) + NH4, Cer(d14:0_17:1) + H, and CL(81:13)-2H (Figure 5; Supplementary Table S1).

### 3.5 Correlation between lipidomics results and phenotypic indices

**Figure 6:**
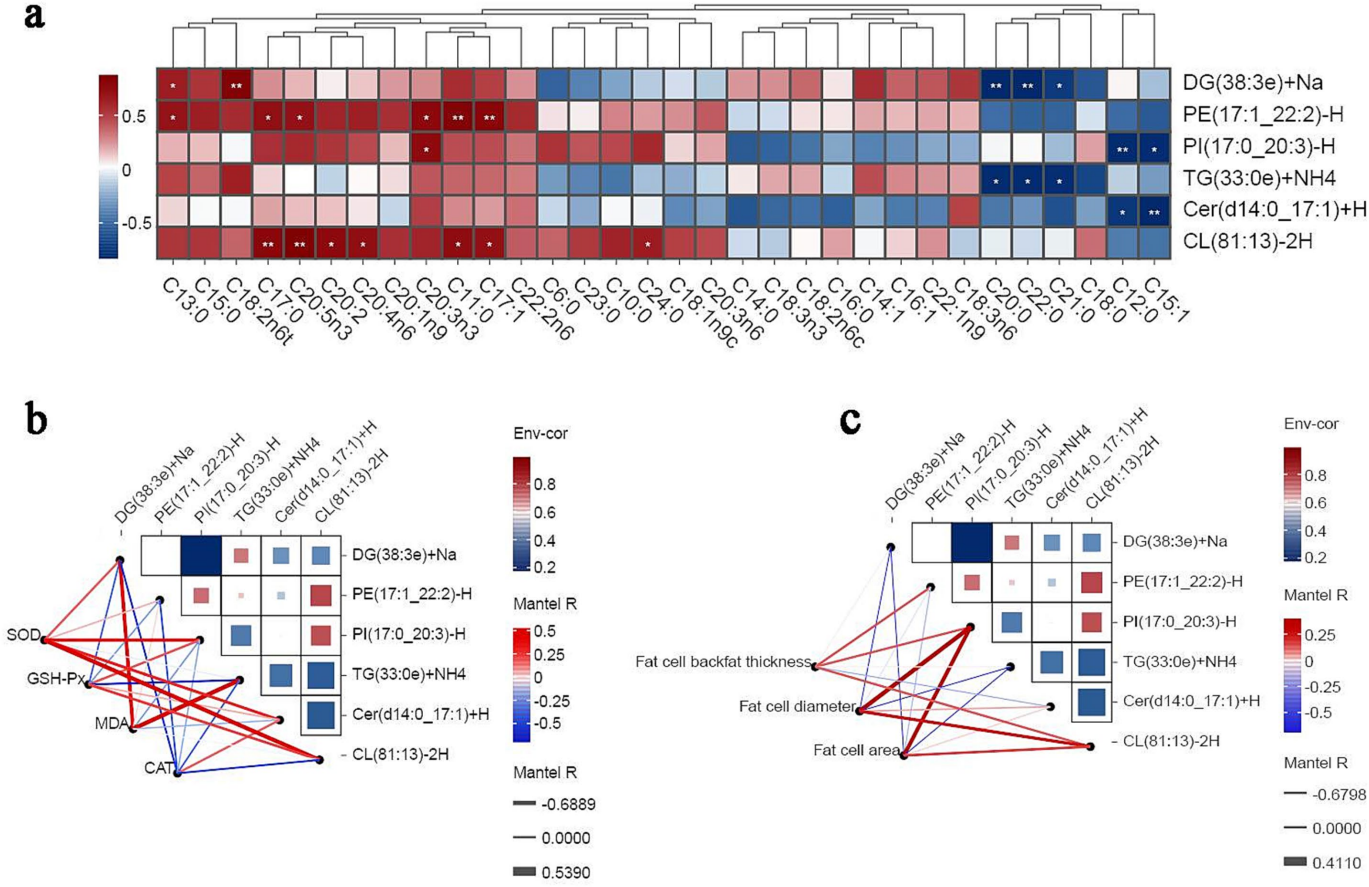
Correlation analysis. In a Spearman’s correlation analysis, blue indicates a negative correlation, red indicates a positive correlation, and the darker the color, the stronger the correlation. Edge width corresponds to the Mantel’s r statistic for the corresponding distance correlation, and edge color denotes statistical significance. * *p* < 0.05. ** *p* < 0.01. *** *p* < 0.001. **(A)** Correlation analysis of lipid molecules with fatty acid content index. **(B)** Correlation analysis of lipid molecules with sebum antioxidant index. **(C)** Correlation analysis of lipid molecules with adipose tissue morphology.

The correlation of different lipid molecules with the abundance of different FAs in subcutaneous fat was analyzed using combined lipidomics–phenotypic indices analysis and Spearman’s correlation coefficients. The results showed that the abundance of the lipid molecule DG(38:3e) + Na was positively correlated with C13:0 and C18:2n6t levels. Meanwhile, the abundance of PE(17:1_22:2)-H was positively correlated with the levels of C13:0, C17:0, C20:5n3, C20:3n3, C11:0, and C17:1, and the abundance of PI(17:0_20:3)-H was positively correlated with the levels of C20:1 and C20:3n3. Additionally, the abundance of CL(81:13)-2H was positively correlated with C17:0, C20:5n3, C:20:2, C20:4n6, C11:0, C17:1, and C24:0 levels (Figure 6A). Antioxidant phenotypic correlation analysis (Figure 6B) revealed that SOD activity was positively correlated with the content of all lipid molecules. Meanwhile, GSH-Px and CAT activity was positively correlated with CL(81:13)-2H and Cer(d14:0_17:1) + H levels, respectively. In addition, the abundance of PI(17:0_20:3)-H was positively correlated with adipocyte diameter and adipocyte area. Finally, the abundance of CL(81:13)-2H was positively correlated with adipocyte diameter, cell area, and backfat thickness (Figure 6C).

### 3.6 Gene expression analysis of adipose tissue

**Figure 7:**
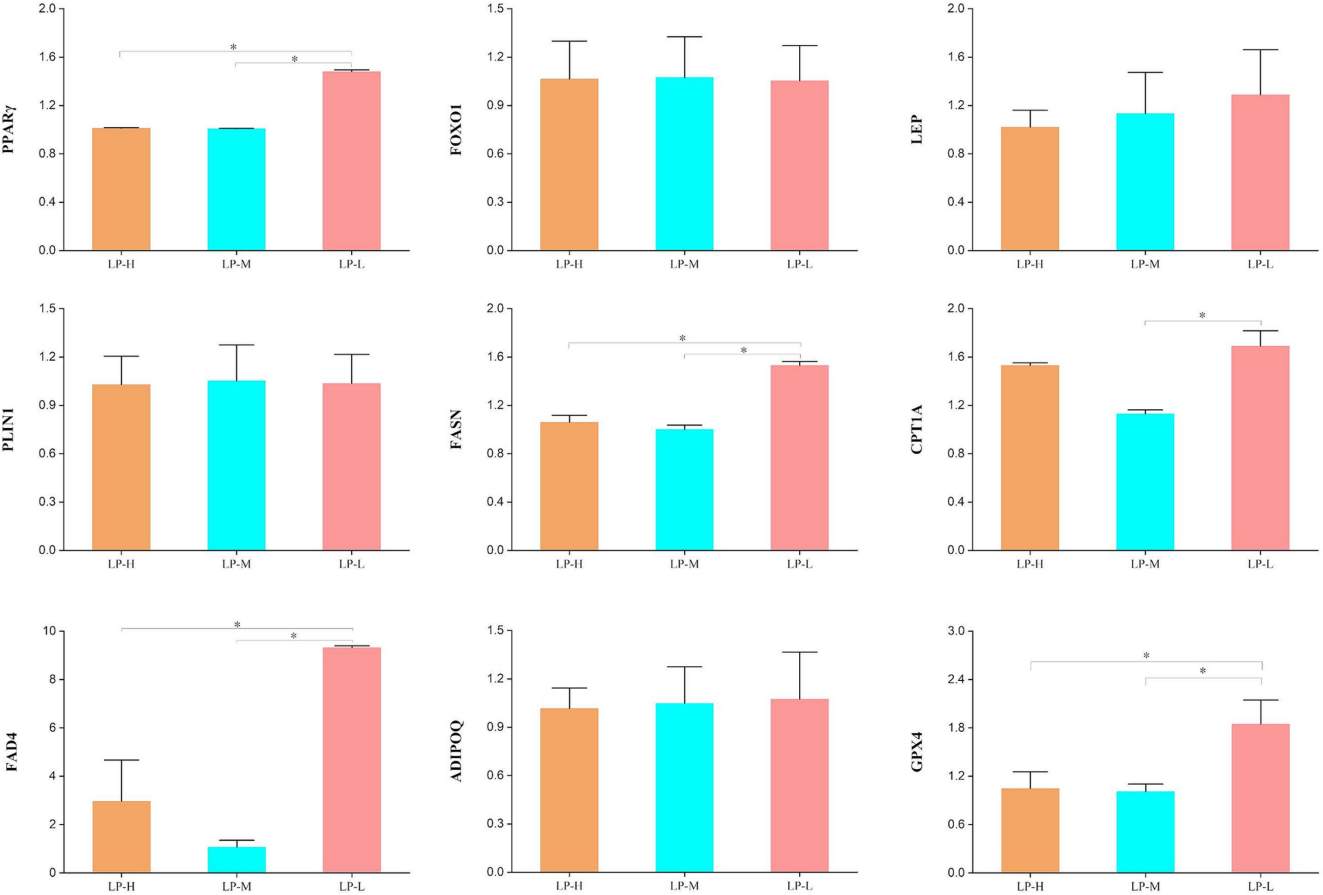
Effect of dietary Lys/Met ratio on the expression of genes related to adipocyte metabolism in subcutaneous fat.

After comparing gene expression levels across the LP-H, LP-M, and LP-L groups receiving diets with different Lys/Met ratios (Figure 7), the LP-L group showed significantly higher expression of *PPARγ, FASN, FABP4, CPT1A,* and *GPX4* compared to the LP-H and LP-M groups (*p* < 0.05). However, there was no significant difference in the expression of *FOXO1*, *LEP*, *PLIN1*, *FASN*, *FAD4* and *ADIPOQ* among the three groups (*p* > 0.05).

## 4 Discussion

Antioxidant enzymes are considered the main defense against cellular damage, and higher activities of CAT, SOD, and GSH-Px can enhance intracellular free radical scavenging (13). MDA is the end product of lipid peroxidation, and elevated MDA concentration typically reflect the lipid peroxidation of cellular membranes, which can lead to impairments in cell structure and function (14). The results of this study showed that the MDA concentration was significantly lower in sheep with a Lys/Met ratio of 1:1 compared to sheep with a Lys/Met ratio of 2:1 and 3:1. This indicates that the addition of Lys/Met at a 1:1 ratio does not cause damage to adipocytes in Tibetan sheep. One possible explanation is that a 1:1 Lys/Met ratio as part of a low-protein diet can enhance antioxidant and cellulase activity in the rumen by modulating the microbiota and metabolism of Tibetan sheep (2). Amylase is an important enzyme through which rumen microorganisms break down starch. When the Lys/Met ratio is 1:1, the concentration of amylase increases, allowing rumen microorganisms to break down starch more efficiently and produce more volatile fatty acids (acetic acid and propionic acid). These volatile fatty acids provide additional energy for the animal, which reduces the body’s reliance on fat reserves. This lowers the metabolic pressure on adipose tissue, reduces lipid peroxidation, lowers MDA concentration, thereby preventing damage to fat cells (15). Meanwhile, GSH-Px is a selenium-containing antioxidant enzyme that protects cells from oxidative damage, particularly the peroxidation of lipids in cell membranes (16). Previous studies have shown that high stocking densities reduce the total antioxidant capacity and GSH-Px activity in animals, but dietary supplementation with Met mitigates this effect (17). In this study, GSH-Px activity was found to be significantly elevated when the Lys/Met ratio was 1:1. The addition of Lys and Met at a ratio of 1:1 could improve immune function and provide protection against free radicals in Tibetan sheep. This could be attributed to the ability of exogenous Met supplementation to increase the elimination of reactive oxygen species (ROS) through Met residues or glutathione synthesis, directly or indirectly affecting the activity of antioxidant enzymes (18). Met contains sulfur atoms and can contribute to the synthesis of glutathione (GSH) through its metabolites, thus increasing the antioxidant capacity of the body (19). This is in agreement with the results reported by Mavrommatis and colleagues (20). Collectively, the findings show that the dietary supplementation of Lys and Met at a ratio of 1:1 increases GSH-Px and SOD levels and decreases MDA concentration in Tibetan sheep.

In domestic animals such as cattle and sheep, fat serves as a major reservoir of energy. Subcutaneous fat is especially important in maintaining body temperature, protecting internal organs, and providing a buffer against external stress (10). In this study, microscopy observations revealed that the adipocytes in the Lys/Met 1:1 group were more neatly arranged, with an overall uniform size, and most of them were rhombic in shape. This rhombic cell morphology could enhance the cell surface area for interactions with other cells or the extracellular matrix, promoting metabolic processes in the adipocytes. Uniformly sized cells enable the maintenance of the integrity and structural stability of adipose tissue, which is critical for its normal function. Adipose tissue is required for regulating the systemic energy balance, glucose homeostasis, immune response, reproduction, and longevity (21). In cases of obesity or metabolic dysfunction, fat cells can become smaller and dysfunctional, potentially inducing chronic low-grade inflammation (22). Thus, the consistency in the size and arrangement of fat cells contributes to efficient energy storage and ensures that energy can be efficiently released when required. The normal morphology and arrangement of adipocytes are associated with a lower risk of metabolic syndrome and can prevent chronic disorders such as cardiovascular disease and diabetes (23). In subcutaneous adipose tissue, neatly aligned adipocytes also distribute mechanical pressure more efficiently and reduce the compression of surrounding tissues and organs. In the present study, the diameter and area of adipocytes were compared among the three groups, and it was found that the Lys/Met 3:1 and 2:1 groups had significantly lower adipocyte diameters and areas than the Lys/Met 1:1 group. Larger adipocytes can store more fat, providing an additional source of energy in times of food shortage or increased energy demand (cold environments or prolonged exercise) (24, 25). As expected, temporal changes in lipolysis per cell were closely correlated with changes in adipocyte volume. We speculate that this may be because diets with Lys/Met ratios of 3:1 and 2:1 promote the expression of lipolysis-related genes. The accelerated rate of lipolysis prevents the synthesized fat from being stored in the body, leading to the rapid breakdown and metabolism of lipid molecules (26). Additionally, there was no significant difference in backfat thickness among the three groups, further indicating that dietary Lys/Met supplementation is a favorable approach for developing lean muscle tissue in Tibetan sheep.

Fatty acid (FA) composition is a crucial factor in evaluating the fat content and healthiness of meat products (27). In this study, we found that different Lys/Met ratios affected the contents of C11:0, C13:0, C15:0, and C20:0 in Tibetan sheep. The Lys/Met ratio of 1:1 reduced the levels of C11:0 and C13:0, which are associated with increased low-density lipoprotein cholesterol (LDL-C) and cardiovascular disease risk (28, 29). This could be due to the more homogeneous nutrient environment and optimized lipid metabolism-related enzyme activities in the 1:1 group, leading to decreased conversion of unsaturated to saturated FAs (30). Additionally, the increased antioxidant capacity in this group may have reduced the accumulation of unhealthy FAs. Interestingly, the C15:0 content increased as the Lys/Met ratio decreased. C15:0 has been shown to target multiple hallmarks of aging, repair mitochondrial function, and reduce the risk of lipid peroxidation (31, 32). C15:0 supplementation has also been found to reduce adiposity, insulin levels, and blood pressure in adolescents (33). However, the significantly higher C20:0 content in the Lys/Met 1:1 group contrasts with findings by Sun et al. (34), who reported that high levels of C20:0 increase oxidative stress and induce apoptosis and inflammation in hepatocytes. This discrepancy may be attributed to differences in FA hydrogenation by rumen microorganisms, as well as variations in animal varieties, environments, and geographical regions. Further research is needed to elucidate the specific mechanisms underlying these effects.

Previous studies have shown that polyunsaturated fatty acids (PUFAs) in feeds have important regulatory effects on intestinal microecology, metabolism, immunity, and reproduction in livestock and poultry (35). In this study, we found that the Lys/Met ratio can affect the accumulation of long-chain unsaturated FAs such as C20:5n3 (EPA) by regulating FA synthesis and metabolic pathways. At a 1:1 Lys/Met ratio, the synthesis of EPA was promoted and its breakdown was reduced. EPA, an omega-3 PUFA, promotes FA *β*-oxidation and ATP production, effectively inhibiting age-related pathological changes and slowing down the aging process (36). Additionally, EPA can regulate serum lipid levels, promoting the elimination of cholesterol and lowering plasma levels of cholesterol, triglycerides, LDL, and VLDL, while increasing HDL levels (37). These findings suggest that a 1:1 Lys/Met ratio may favor the down-regulation of blood glucose and cholesterol levels, improving cardiovascular health and potentially enhancing meat flavor. Interestingly, the levels of C17:1 and C20:2 were also affected by different Lys/Met ratios. C17:1, a ginkgo FA, can regulate oligofructose (FOS) content, which positively regulates fat deposition, lipid metabolism, and egg quality in hens (38). The elevated C17:1 content in the Lys/Met 1:1 group could be due to the favorable FA flow in the cell membrane, promoting the activity of specific FA synthase (FAS) and increasing C17:1 accumulation (39). C20:2, a long-chain PUFA (LC-PUFA), was significantly elevated in the 1:1 Lys/Met group, suggesting that this ratio enhances the efficiency of linoleic acid (LA) and *α*-linolenic acid (ALA) conversion, modulating key lipid metabolic pathways (40). The increase in C20:2 may provide physiological benefits, such as enhanced inflammatory response regulation, immune response, neurotransmitter synthesis, cholesterol metabolism, and maintenance of membrane phospholipid structure (41, 42). Furthermore, C20:2 may play an immunomodulatory and supportive functional role in brain and retina health (43, 44).

Lipidomics, a branch of metabolomics, utilizes GC–MS to accurately quantify lipid molecules, enabling the exploration of the intrinsic link between lipid molecules and various phenotypic features (45). In this study, 39 lipid subclasses encompassing 2,605 lipid molecules were identified under the positive and negative ion models. The typical lipid molecules associated with the phenotype (Antioxidant index and fatty acid content) were TG(33:0e) + NH4, PE(17:1_22:2)-H, PI(17:0_20:3)-H, DG(38:3e) + Na, Cer(d14:0_17:1) + H, and CL(81:13)-2H. The lipid heterogeneity across different lipid molecules in the subcutaneous adipose tissues of different groups was identified by OPLS-DA analysis.

In this study, we found that TG(33:0e) + NH4, a triglyceride synthesized via the esterification of glycerol with three FAs, was up-regulated at a Lys/Met ratio of 1:1. This triglyceride promotes adipocyte differentiation by activating specific nuclear receptors, such as PPARγ, which regulates adipose tissue morphology and enhances FA metabolism (46). TG(33:0e) + NH4 also reduces inflammation and oxidative stress in the context of RAGE inhibition. However, during *β*-oxidation, this FA provides energy and regulates lipid metabolism homeostasis (47). Correlation analysis revealed a positive association between TG(33:0e) + NH4 and MDA, suggesting that high triglyceride levels may promote lipid peroxidation (48, 49). PE(17:1_22:2)-H, a phosphatidylethanolamine and a main component of the cell membrane, protects against oxidative damage by maintaining membrane fluidity and regulating the redox balance (50, 51). The elevated PE(17:1_22:2)-H content in the Lys/Met 1:1 group could be attributed to the optimized phospholipid synthesis pathways and enhanced antioxidant capacity provided by this amino acid ratio. PI(17:0_20:3)-H, a phosphatidylinositol, plays a crucial role in the PI3K/AKT signaling pathway. A Lys/Met ratio of 1:1 may activate this pathway more efficiently, increasing PI(17:0_20:3)-H levels (52). PI(17:0_20:3)-H can be phosphorylated by PI3K, generating PIP3, which activates AKT and promotes SREBP-1c activity, directly involved in the transcription of the FAS gene (53–55). The positive correlation between PI(17:0_20:3)-H and FA C20:3n3 levels suggests that common metabolic pathways may contribute to the synthesis of both compounds (56).

DG(38:3e) + Na, a diacylglycerol and product of triglyceride catabolism, plays a crucial role in cell signaling by activating protein kinase C (PKC) in adipocytes. The PKC signaling pathway regulates adipocyte differentiation and lipid metabolism, affecting adipose tissue morphology (57). DG(38:3e) + Na also facilitates fat breakdown during catabolism by interacting with enzymes such as triglyceride lipase (ATGL) and hormone-sensitive lipase (HSL) (58). In this study, DG(38:3e) + Na was elevated when the Lys/Met ratio was 1:1, possibly due to Lys activating phospholipase C (PLC), which promotes the generation of DG (57). Correlation analysis showed a positive association between DG(38:3e) + Na and the fatty acid C18:2n-6, suggesting that they can regulate each other during fat metabolism, signaling, energy homeostasis, and inflammatory responses (59). Cer(d14:0_17:1) + H, a ceramide and sphingolipid, is an important component of cell membranes and regulates cell signaling, stress response, and apoptosis (60). Cer(d14:0_17:1) + H levels were up-regulated in the Lys/Met 1:1 ratio group, leading to more complete cellular organization. This lipid molecule also plays a key role in regulating lipid metabolism, adipocyte differentiation, and lipid storage, while enhancing cellular antioxidant capacity (61). Under hypoxic conditions, Cer(d14:0_17:1) + H can increase cellular energy supply and antioxidant capacity by promoting glycolysis (62). Furthermore, Cer(d14:0_17:1) + H can indirectly affect fatty acid metabolism by regulating fatty acid oxidation and intracellular signaling pathways, modulating fatty acid synthesis and catabolism in response to nutrient status (63).

CL(81:13)-2H (cardiolipin) is a unique non-bilayer-forming glycerophospholipid that is found in the membranes of prokaryotes and the mitochondrial membranes of eukaryotes (50). CL(81:13)-2H is essential for oxidative phosphorylation, which affects ATP production. When CL(81:13)-2H levels decrease, mitochondrial function is compromised, leading to impaired energy metabolism. This, in turn, results in inadequate FA oxidation, thereby affecting the metabolism of adipose tissue (64). In this study, the increase in CL(81:13)-2H in the Lys/Met 1:1 ratio group could be attributed to the positive effect of Lys on the synthesis of carnitine, which is essential for FA *β*-oxidation. This increase could enhance mitochondrial energy metabolism, thereby promoting CL(81:13)-2H biosynthesis (65). The 1:1 ratio of Lys and Met may provide cells with sufficient metabolic precursors to optimize mitochondrial function and CL(81:13)-2H synthesis (66). This suggests that a proper supply of amino acids may enhance mitochondrial function and promote the increase in CL(81:13)-2H content. Spearman’s correlation analysis in the present study revealed that the content of CL(81:13)-2H was positively correlated with the content of FA C20:5n3 and the antioxidant indicator SOD. C20:5n3 upregulates nuclear factor erythroid 2-related factor 2 (Nrf2), a transcription factor that is commonly expressed in the cardiovascular system. It also decreases the sensitivity of cardiomyocytes to ROS (67). Additionally, Nrf2 increases the expression of antioxidant enzyme-related genes, including heme oxygenase-1, thioredoxin reductase 1, ferritin light chain, ferritin heavy chain, and manganese superoxide dismutase (68). Zhou et al. demonstrated that during ischemia–reperfusion injury (IRI), C20:5n3 increases the levels of antioxidant enzymes, such as GSH-Px and SOD, preventing further tissue damage (69). Thus, the evidence indicates that Lys and Met, as basic units of protein synthesis, mediate the effect of C20:5n3 on CL(81:13)-2H and antioxidant enzymes. The results of the present study are consistent with the above findings.

In this study, we identified nine candidate genes involved in the regulation of subcutaneous fat generation, differentiation, antioxidant activity, and FA metabolism—namely, *PPARγ, ADIPOQ, FAD4, FASN, FOXO1, GPX4, LEP, PLIN1,* and *CPT1A*. The master regulator *PPARγ* orchestrates adipocyte differentiation through a cascade of transcriptional events, not only promoting adipogenesis but also establishing a metabolically favorable environment that prevents lipotoxicity in non-adipose tissues (70). This process is intimately coupled with *FASN*-mediated *de novo* lipogenesis, where FAS serves as a rate-limiting enzyme in the conversion of acetyl-CoA to long-chain fatty acids (71). The upregulation of *PPARγ* and *FASN* promotes the synthesis of triglycerides and diglycerides, contributing to increased fat storage, which manifests as larger adipocytes and greater lipid accumulation in adipose tissues. This response represents an adaptive strategy through which Tibetan sheep augment fat reserves and improve cold tolerance in extreme environments (72). *FAD4* participates in lipid synthesis and metabolism, acting as a crucial element of FA desaturation. Additionally, it contributes to membrane fluidity in adipocytes and influences the composition and quality of subcutaneous fat (73). Meanwhile, carnitine palmitoyltransferase 1A (*CPT1A*) plays a pivotal role in the mitochondrial *β*-oxidation of FAs, ensuring the efficient utilization of subcutaneous fat for energy production, particularly during durations of heightened metabolic demand (74). Furthermore, glutathione peroxidase 4 (*GPX4*) provides protective effects in subcutaneous adipose tissue by mitigating oxidative stress and preventing lipid peroxidation (75). Our findings reveal a particular vulnerability of PE and PI phospholipids to oxidative damage, highlighting the critical importance of *GPX4* upregulation in maintaining membrane integrity. The protection of specific phospholipid species, such as PE(17:1_22:2)-H and PI(17:0_20:3)-H, suggests a targeted approach to membrane stabilization that is essential for proper adipocyte function (76). This protective mechanism becomes particularly crucial under conditions of metabolic stress, where maintaining adipocyte homeostasis is essential for proper tissue function (77). The observed 1:1 Lys/Met ratio appears to create an optimal amino acid environment that synchronizes multiple metabolic pathways. The coordinated upregulation of *PPARγ* and *FASN* suggests that amino acid balance serves as a metabolic signal, fine-tuning both adipogenesis and lipid accumulation in Tibetan sheep. Furthermore, the enhanced expression of *CPT1A* and *GPX4* indicates that proper amino acid ratios can simultaneously optimize both energy utilization and cellular defense mechanisms in subcutaneous adipose tissue. This intricate relationship between dietary amino acid composition and gene expression patterns reveals a sophisticated regulatory network where nutritional inputs are translated into coordinated metabolic responses, ultimately determining the efficiency of subcutaneous fat storage and utilization.

## 5 Conclusion

In this study, a 1:1 Lys/Met ratio improved antioxidant capacity, increased unsaturated FA levels, and positively regulated the expression of genes related to adipocyte differentiation, lipid storage, FA metabolism, and antioxidant defense in the subcutaneous adipose tissue of Tibetan sheep. The associations among the phenotypes, differential gene expression, and lipid metabolites observed in this study suggest that the 1:1 Lys/Met ratio can improve adipocyte function and lipid metabolism in Tibetan sheep. These findings provide a theoretical basis for the development of more rational and effective feeding and dietary supplementation strategies for optimizing the quality and nutritional value of meat products from Tibetan sheep.

## Data Availability

The datasets presented in this study can be found in online repositories: https://www.cncb.ac.cn/, login number OMIX008720, project number PRJCA035194.
